# Mutual Information Better Quantifies Brain Network Architecture in Children with Epilepsy

**DOI:** 10.1155/2018/6142898

**Published:** 2018-10-22

**Authors:** Wei Zhang, Viktoria Muravina, Robert Azencott, Zili D. Chu, Michael J. Paldino

**Affiliations:** ^1^Department of Radiology, Texas Children's Hospital, 6701 Fannin St., Houston, TX, USA; ^2^Outcomes and Impact Service, Texas Children's Hospital, 6701 Fannin St., Houston, TX, USA; ^3^Department of Mathematics, University of Houston, 3507 Cullen Blvd, Houston, TX, USA; ^4^Department of Radiology, Baylor College of Medicine, One Baylor Plaza-BCM360, Houston, TX, USA

## Abstract

**Purpose:**

Metrics of the brain network architecture derived from resting-state fMRI have been shown to provide physiologically meaningful markers of IQ in children with epilepsy. However, traditional measures of functional connectivity (FC), specifically the Pearson correlation, assume a dominant linear relationship between BOLD time courses; this assumption may not be valid. Mutual information is an alternative measure of FC which has shown promise in the study of complex networks due to its ability to flexibly capture association of diverse forms. We aimed to compare network metrics derived from mutual information-defined FC to those derived from traditional correlation in terms of their capacity to predict patient-level IQ.

**Materials and Methods:**

Patients were retrospectively identified with the following: (1) focal epilepsy; (2) resting-state fMRI; and (3) full-scale IQ by a neuropsychologist. Brain network nodes were defined by anatomic parcellation. Parcellation was performed at the size threshold of 350 mm^2^, resulting in networks containing 780 nodes. Whole-brain, weighted graphs were then constructed according to the pairwise connectivity between nodes. In the traditional condition, edges (connections) between each pair of nodes were defined as the absolute value of the Pearson correlation coefficient between their BOLD time courses. In the mutual information condition, edges were defined as the mutual information between time courses. The following metrics were then calculated for each weighted graph: clustering coefficient, modularity, characteristic path length, and global efficiency. A machine learning algorithm was used to predict the IQ of each individual based on their network metrics. Prediction accuracy was assessed as the fractional variation explained for each condition.

**Results:**

Twenty-four patients met the inclusion criteria (age: 8–18 years). All brain networks demonstrated expected small-world properties. Network metrics derived from mutual information-defined FC significantly outperformed the use of the Pearson correlation. Specifically, fractional variation explained was 49% (95% CI: 46%, 51%) for the mutual information method; the Pearson correlation demonstrated a variation of 17% (95% CI: 13%, 19%).

**Conclusion:**

Mutual information-defined functional connectivity captures physiologically relevant features of the brain network better than correlation.

**Clinical Relevance:**

Optimizing the capacity to predict cognitive phenotypes at the patient level is a necessary step toward the clinical utility of network-based biomarkers.

## 1. Introduction

Computational methods now have the capacity to model the cerebral network at the whole-brain scale [1]. In this context, the brain is represented as a collection of anatomical elements, or nodes; connections between pairs of nodes, referred to as edges, are then measured noninvasively. Once constructed, the organization of the resulting network can be quantified according to graph theoretical principles [2]. These techniques offer the potential to capture physiologically relevant architectural features of the cerebral network [3]. Resting-state functional MRI (rs-fMRI), a sequence that measures the blood oxygen level-dependent (BOLD) signal over time, is one method by which edges in the brain network can be quantified. Elements of the brain that interact to support a given function continue to exhibit similar BOLD fluctuations at rest [4]. Hence, the strength of a connection between each pair of nodes can be inferred from the similarity of their BOLD signal time courses. As this sequence is task free, it offers the potential to measure the functional status of children who are too young or too impaired to cooperate with traditional functional imaging. These attributes point to the potential for resting-state approaches to deliver new clinical tools, especially in disorders of the brain that emerge from reorganization of the cerebral network such as epilepsy [3]. Recent work has demonstrated the potential of network metrics derived from rs-fMRI to provide clinically meaningful markers of cognitive function in adults [5, 6], in healthy children [7], and in children with focal epilepsy [5–8]. Despite this promise, exactly how neuronal interaction across the cerebrum is reflected by these spontaneous fluctuations in the BOLD signal—and therefore how to best measure similarity in BOLD time courses—is yet to be determined.

The most commonly used measure of functional connectivity in resting-state studies is the Pearson correlation coefficient, defined as the linear covariance of two variables divided by the product of their standard deviations. The Pearson correlation coefficient is simple to calculate and facilitates communication among researchers of diverse disciplines. However, a critical assumption inherent to the use of correlation in the resting state—that the physiologically relevant information about interactions between two discrete brain regions is reflected by a linear relationship between the values of their respective BOLD signals at the same time—may not be valid. In particular, recent studies have shown that nonlinearities inherent to resting-state acquisitions, predominantly hemodynamic in origin, affect both the timing and the amplitude of the measured BOLD signal [9]. As a result, relationships between time series are influenced by the profile of temporal interactions rather than by zero-lag interactions alone [10, 11]. Furthermore, recent work has suggested that nonlinear relationships may play an even more prominent role in the connectivity of pathologic tissues [12]. Beyond issues of linearity, there is a great deal of uncertainty in terms of how the true neuronal interactions we hope to measure are represented by fluctuations in blood flow (BOLD); this challenge highlights the importance of generality [13]. Mutual information is an alternative measure of similarity that quantifies in a very general way how much one random variable tells us about another. It is a dimensionless quantity and can be thought of as the reduction in uncertainty about one variable given knowledge of another. Mutual information has been shown to outperform other methods for characterizing association between time series in simulated networks, in part for its generality and equitability [10, 13]. It has also been shown to provide a repeatable estimate of network connectivity in the brains of normal subjects [10]. Little data exist, however, regarding the importance of nonlinear association within resting-state networks with regard to the emergence of cognitive dysfunction. We therefore sought to compare brain networks constructed from mutual information to those based on correlation in terms of their capacity to support patient-level inferences on the relationship between brain network architecture and brain function.

## 2. Methods

### 2.1. Patients

The HIPAA-compliant study was approved by a local institutional review board. Informed consent was waived. Patient medical records were retrospectively reviewed to identify patients with the following inclusion criteria: (1) pediatric age group (21 years of age or younger); (2) a clinical diagnosis of focal epilepsy; (3) an available 3 tesla MR imaging of the brain, including an rs-fMRI sequence; and (4) full-scale intelligence quotient (IQ) using an age-appropriate Wechsler Intelligence Scale measured by a pediatric neuropsychologist within 3 months of the MR imaging. The above-defined cohort was refined by applying the following exclusion criteria: (1) any brain operations performed prior to the MR imaging or (2) having poor image quality due to either motion or other artifacts.

Imaging was performed from January 2013 to June 2015. Thirty-four patients met the inclusion criteria. Ten were excluded on the basis of prior brain surgery. Twenty-four patients (age range: 8–18 years; median: 13.4; 12 (46%) females) made up the final cohort. Of this cohort, 5 patients had structurally normal brains and 19 patients had demonstrable structural abnormalities at MRI, including focal cortical dysplasia (*n*=8), mesial temporal sclerosis (*n*=5), low-grade tumor (*n*=4), and a single epileptogenic tuber in the setting of tuberous sclerosis (*n*=2). An age-appropriate version of the Wechsler intelligence test was successfully administered in all patients; full-scale intelligence quotient in the cohort ranged from 52 to 129 (median: 91).

### 2.2. MR Imaging

All imaging procedures were performed on a 3 tesla Achieva system (Philips, Andover, Massachusetts) with a 32-channel phased array coil. The following sequences were obtained: (1) structural images: sagittal volumetric T1-weighted images (repetition time (TR)/echo time (TE): 7.2 ms/2.9 ms; 1 acquisition; flip angle: 7°, inversion time: 1100 ms; field of view (FOV): 22 cm; voxel size (mm): 1 × 1 × 1), and (2) resting-state fMRI: axial single-shot echo planar imaging (EPI) fMRI (TR/TE (ms): 2000/30; flip angle: 80°; 1 acquisition; FOV: 24 cm; voxel (mm): 3 × 3 × 3.75; 300 volumes (duration: 10 minutes)) performed in the resting state. Patients were instructed to lie quietly in the scanner with their eyes closed. All images were visually inspected for artifacts, including susceptibility and subject motion.

### 2.3. Image Processing and Analysis

The processing pipeline was implemented using MATLAB scripts (version 7.13; MathWorks, Inc.) in which adapter functions were embedded to execute FreeSurfer reconstruction (version 5.3.0; http://surfer.nmr.mgh.harvard.edu) and several FMRIB software library (FSL) suite tools [14]. Details regarding this pathway have been previously described [3, 8]. A brief summary is provided here.

#### 2.3.1. Network Node Definition

The reference space was created from images of one patient in our database, who had no visible abnormality and with optimal registration to the MNI space [15]. Structural imaging data for each patient were aligned to a standard reference template (MNI152) using the FSL's nonlinear registration algorithm [14, 16]. Nodes in the network were defined on the template according to parcellation of whole-brain gray matter. First, FreeSurfer reconstruction of cerebral cortical surfaces was performed on the T1 structural image. This processing stream includes motion correction, skull stripping, intensity normalization, segmentation of white matter and gray matter structures, parcellation of the gray matter and white matter boundary, and surface deformation following intensity gradients which optimally place the gray matter/white matter and gray matter/cerebrospinal fluid borders [17, 18]. The pial and gray white surfaces were visually inspected using the Freeview software for accurate placement.

**Figure 1:**
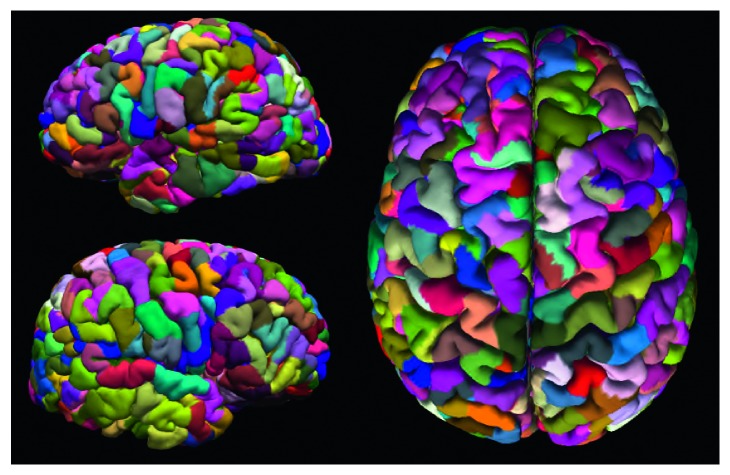
Final parcellation with a size threshold of 350 mm^2^, resulting in 780 nodes.

Next, a self-developed MATLAB program was applied to the FreeSurfer output to further subdivide the 75 standard gray matter parcels according to their surface area. During this process, each parcel was iteratively divided into two new parcels of equal size until the surface area of each parcel (as defined on the FreeSurfer gray-white surface mesh) was less than a predetermined threshold value. Networks were constructed with a size threshold of 350 mm^2^. The final parcellation contained 780 nodes (Figure 1). Each surface parcel was then converted into a volume mask of gray matter at that region to form a node on the network. All nodes defined in the reference space were transformed into each individual patient's space by applying the nonlinear transformation matrix (12 degrees of freedom) obtained during registration.

#### 2.3.2. FMRI Data Preprocessing

The first 5 volumes in each resting-state functional datum were removed to allow magnetization to reach equilibrium. Standard preprocessing and independent component analysis (ICA) of the functional datasets were performed using FSL MELODIC [14], consisting of motion correction, interleaved slice timing correction, brain extraction, spatial smoothing with a Gaussian kernel full width at half maximum of 5 mm, and high-pass temporal filtering equivalent to 100 seconds (0.01 Hz). Noise related to motion and other physiologic nuisance was addressed according to an independent component analysis technique [19]. Nonsignal components were removed manually by an expert operator with 6 years of experience using independent component analysis in this patient population. Although the optimal strategies for noise removal in fMRI are debatable [20, 21], an independent component analysis was selected because it has been shown to minimize the impact of motion on network metrics while, at the same time, decreasing the loss of temporal degree of freedom and preserving the signal of interest across a variety of resting-state datasets [21]. Affine boundary-based registration as implemented in FSL FLIRT was then used to align the preprocessed functional image volumes for each patient to that individual's structural T1 dataset using linear registration. The inverse transformation matrix was calculated in this step and subsequently used to transform all masks from structural to functional space. Mean BOLD signal time series were then computed for each node.

#### 2.3.3. Network Edge Definition

The strength of an edge (connection) between 2 nodes was defined in two ways: (1) the absolute value of the Pearson correlation coefficient between their BOLD time series and (2) the mutual information calculated based on the following method.

For two discrete random variables *X* and *Y*, their mutual information takes the following form:

(1) $$\begin{array}{c} \text{MI}\left(X,Y\right)=\underset{x\in S_{x}}{\sum }\underset{y\in S_{y}}{\sum }p\left(x,y\right)\;\log\;\left(\frac{p\left(x,y\right)}{p\left(x\right)p\left(y\right)}\right), \end{array}$$

where *S*_*x*_ and *S*_*y*_ are possible values of *X* and *Y*, *p*(*x*, *y*) is the probability that the pair (*X*, *Y*) takes values *x* in *S*_*x*_ and *y* in *S*_*y*_, and *p*(*x*) and *p*(*y*) are two marginal probabilities of *X* and *Y*. For a pair of time series taking small number of values, the probability functions *p*(*x*), *p*(*y*), and *p*(*x*, *y*) are estimated by frequency counts of the values *x* and *y* appeared in the time series. Applying this formula to continuous time series seen in most studies requires a grid to discretize the continuous space into small boxes. The probability functions *p*(*x*), *p*(*y*), and *p*(*x*, *y*) then are the frequency counts of values within the boundaries of a box centered at *x* and *y*. The boundaries and the resolution of the grid affect the value of mutual information. To avoid the ambiguity of choosing a grid, we take the largest mutual information of all possible grids of a predetermined resolution. This maximization applied in the mutual information calculation shares the same principal as the maximal information coefficient (MIC), where the maximization is taken over all grids up to a maximal resolution [13]. It is computationally impossible to search all grids for over 300 thousand pair time series of a single patient in our study however. Therefore, the resolution of the grids had to be predetermined. By testing data of several randomly selected patients, the 3-by-3 grids were chosen in the study as they provided similar mutual information compared to finer grids but required much shorter computation time. The boundaries of 3 bins on *x*- or *y*-axis of 3-by-3 grids were determined by 4 values. The two ends are min and max of a time series. The middle two values were determined by mean ± a multiple of standard deviation of the time series. We chose 5 values for the multiple, which yielded 25 possible choices for a pair of the middle two values, 25 choices for 3 bins on *x*- or *y*-axis, and 125 choices for 3-by-3 grids for a pair of time series.

### 2.4. Graph Construction and Network Metric Calculation

Two weighted, undirected connection matrices of each patient were constructed, named as “Pearson and mutual information graphs,” consisting, respectively, of the pairwise Pearson correlations and the mutual information between BOLD time series over all network nodes. The following topologic properties were calculated by using MATLAB scripts provided in the Brain Connectivity Toolbox (https://sites.google.com/site/bctnet/): clustering coefficient, modularity, characteristic path length, and global efficiency. A short description of each metric is provided in Table 1.

Clustering coefficient and modularity are metrics that measure the brain's tendency to segregate into relatively independent, local neighborhoods. In other words, these measures reflect the ability of the brain to process specialized functions within highly interconnected functional subnetworks. Characteristic path length and global efficiency measure the global integration of the brain. A short characteristic path length or a high global efficiency indicates that information can be integrated easily across the brain.

### 2.5. Statistical Analyses

Statistical testing was performed using SAS version 9.3 and R language version 3.4.0 (R Foundation for Statistical Computing, Vienna, Austria). The primary endpoint was the predictive value of the 4 output metrics of the brain network architecture (derived from either Pearson or mutual information graphs) with respect to individual intelligence. This multivariate analysis was accomplished using a random forest approach, which has been previously described in detail in [22]. In short, this ensemble learning method operates by constructing a multitude of decision trees during training and outputting the mean of predictions from individual trees. It is based on bootstrap aggregating, or bagging, in which numerous models are fitted during individual bootstrap sampling and then combined by averaging. During training, approximately one-third of the cohort is omitted at random from the training set—this omitted portion of the dataset is considered “out of bag.” The IQ of each individual held out of bag is then predicted based on the “learned” model. Prediction accuracy for the out-of-bag cohort was quantified in two ways: (1) mean absolute error and (2) fractional variation explained [23]. To be specific, the random forest algorithm was given access to only the four network metrics and no other patient information during this analysis. The absolute errors of predictions from Pearson graphs were compared to those from mutual information graphs using the Wilcoxon signed rank test. All random forest models were run 500 times to obtain the 95% confidence interval (CI) for fractional variation explained.

The random forest algorithm was also used to measure the independent contribution of individual network metrics to the prediction of IQ. In other words, it measures the association of each variable after accounting for all other variables. This contribution is estimated for each variable by measuring the error for IQ prediction in the out-of-bag cohort compared with the error that results when that particular variable is negated during bagging.

Connections from a mutual information graph were compared to those from the corresponding Pearson graph through a scatter plot of each patient. Differences in network metrics computed on Pearson versus mutual information graphs were assessed using the Wilcoxon signed rank test. Relationships between Pearson-derived network metrics and mutual information-derived network metrics were also quantified using the Pearson correlation coefficient. The Pearson graph was chosen to measure this association since monotonic relationships were expected. Finally, the univariate association of each metric with IQ was measured in a univariate analysis by the Spearman correlation coefficient.

## 3. Results

### 3.1. Association between Pearson and Mutual Information Graphs

**Figure 2:**
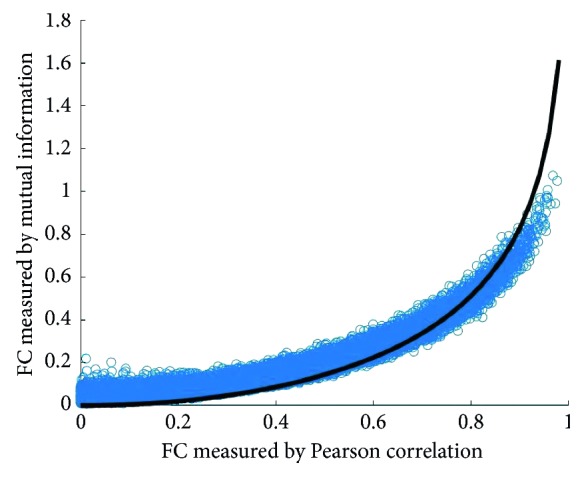
Scatter plot of Pearson and mutual information connections in a representative patient. Each blue dot corresponds to an edge between two nodes in the graph. The black reference line is the function *−*1/2 log (1−*r*^2^), the relationship between mutual information and Pearson correlation when the data are jointly Gaussian.

A representative example of a scatter plot of Pearson versus mutual information connections of one patient is provided in Figure 2. The reference line on the graph is *−*1/2 log (1−*r*^2^), which is the relationship between mutual information and Pearson correlation if the joint distribution is Gaussian [24]. Deviation of our data from the reference line, reflecting nonlinear relationships between resting-state time series, was observed in all patients (data not shown).

**Figure 3:**
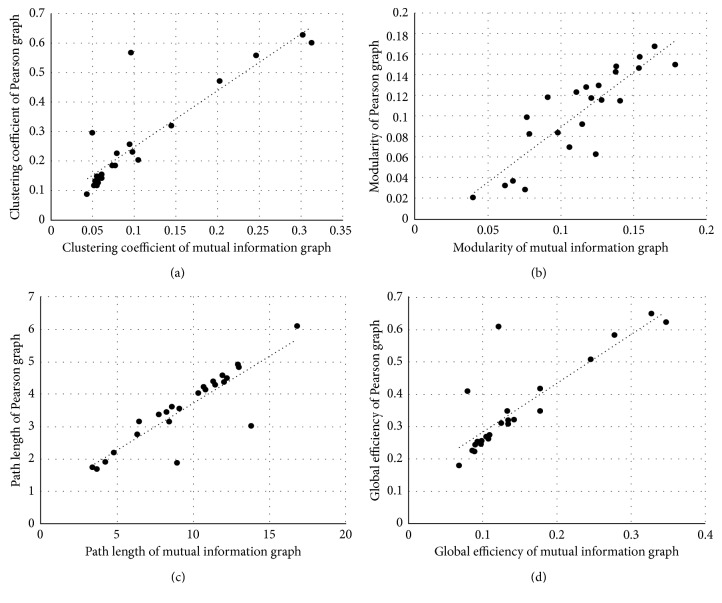
Network metrics derived from Pearson graphs versus those from mutual information graphs: (a) clustering coefficient (*r* = 0.88, *p* < 0.001); (b) modularity (*r* = 0.86, *p* < 0.001); (c) path length (*r* = 0.88, *p* < 0.001); (d) global efficiency (*r* = 0.84, *p* < 0.001).

Network metrics derived from Pearson graphs versus those from the mutual information graphs for each patient are presented in Figure 3. Although association between the Pearson and mutual information graph metrics was generally high-correlation coefficients ranging from 0.84 to 0.88, differences were apparent (Table 2). On average, clustering coefficient and global efficiency became smaller when computed on the mutual information graph. By contrast, path length tended toward higher values under the mutual information graph. Notably, modularity was not statistically different between the graphs (Table 2).

### 3.2. Network Architecture and Intelligence

**Figure 4:**
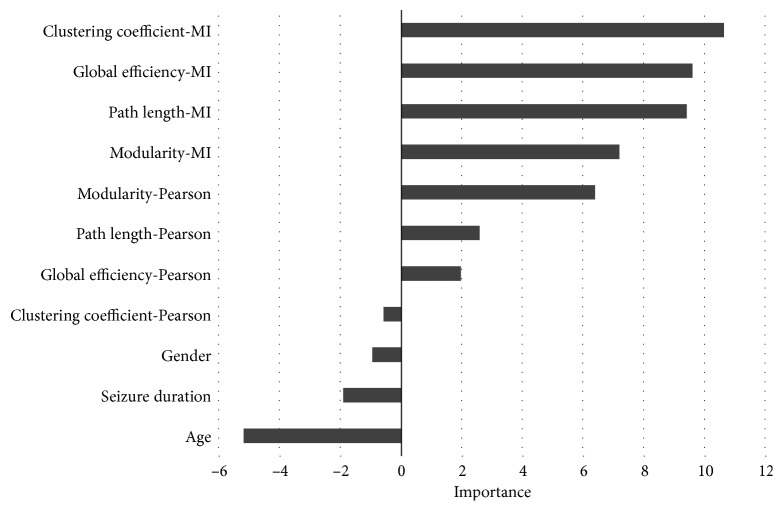
Independent contribution of individual network metrics to IQ prediction by the random forest model.

Univariate correlation of the mutual information and Pearson-derived graph metrics with subject IQ is presented in Table 3. For most metrics, the association with patient IQ was greater when computed on the mutual information graph. Using a multivariate approach, mutual information graph metrics made the dominant contribution to subject IQ prediction by the random forest model (Figure 4).

**Figure 5:**
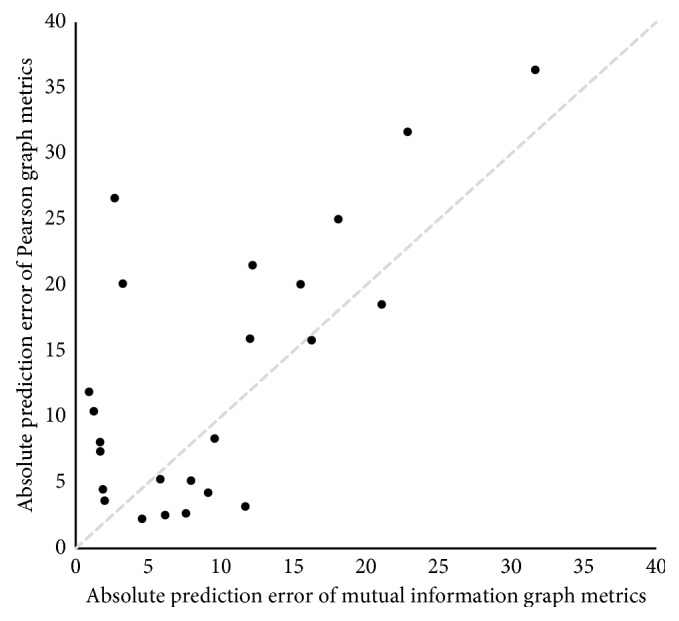
Comparison of absolute errors of “out-of-bag” IQ predictions based on metrics derived from Pearson and mutual information graphs. The gray dash reference line is where the two errors are equal.

Accuracy of the machine learning algorithm's prediction of IQ based on network metrics is presented in Table 4. Metrics derived from mutual information graphs demonstrated a significantly higher predictive value compared to that of the Pearson graph. The relationship between the magnitudes of prediction error for the two methods is demonstrated graphically in Figure 5.

## 4. Discussion

We evaluated two measures of association—the Pearson correlation and mutual information—that are commonly used to infer connectedness in brain networks constructed from resting-state functional MRI. We specifically assessed the impact of these measures on output metrics of the global brain architecture in terms of their capacity to support the prediction of global intelligence in children with focal epilepsy. We report that measuring brain network edges using mutual information significantly outperformed the use of the Pearson correlation in this setting.

Higher-order functions of the human brain are not accomplished by individual functional centers compartmentalized to a particular region of cortex. Rather, they emerge from parallel processing within subspecialized, but distributed, functional systems. The ability to decode these neuronal interactions, particularly as they relate to the emergence of brain function, has become a major focus in current neuroimaging research. Resting-state functional MRI is one modality that has been used extensively as a surrogate for connectedness in the human brain. A significant body of work now exists in support of its capacity to probe physiologically meaningful features of the human brain in a diversity of settings [4]. For example, studies have demonstrated an abnormal network architecture in a variety of disease states, including those with prominent cognitive dysfunction [25–30]; network reorganization has also been observed in adults [31–33] and children [29, 34] with focal epilepsies. Beyond group-level comparisons, a relationship of brain network features quantified by graph theory with intelligence has been demonstrated in many populations, including healthy adults [5, 6, 35], healthy children [7], normal aging [36], Alzheimer's disease [37, 38], autism [39], and epilepsy [8]. Given this capacity of resting-state networks to capture interindividual phenotypic variance in brain function, there is great interest in the development of subject-level markers that could be used to guide patient care [3, 8]. Despite this promise, exactly how neuronal interaction across the cerebrum is reflected by spontaneous fluctuations in the BOLD signal—and therefore how to best measure similarity in BOLD time courses—is yet to be determined. We observed that metrics of the network architecture computed on mutual information graphs outperformed network metrics based on the Pearson correlation in terms of the ability to predict subject-level intelligence in a cohort of children with epilepsy.

We observed that graph-based metrics from Pearson and mutual information graphs were relatively similar and demonstrated high linear correlation. Although this finding is consistent with prior work demonstrating only a small contribution of nonlinear associations to the rs-fMRI time series [24], we found that this “small amount” made a significant difference in terms of subject-level prediction in this population. Interestingly, we also observed larger non-Gaussian dependencies among the time series than what has been reported in a healthy adult population [24]. This idea is consistent with the work by Rummel et al. who used a uniform surrogate-based approach to study interrelations that significantly exceed linear correlation in EEG data of epilepsy patients [12]. They observed that nonlinearity occurred predominantly for epileptogenic tissue as well as during epileptic seizures [12]. Our results align with these studies, suggesting that the dynamics of the abnormal brain may be more complex than those of normal brains and that nonlinear associations may be more prevalent. Therefore, a general measure of brain interactions may be more important when analyzing a disease population.

Our results are in line with the previous work that has used mutual information to quantify network edges. Reshef et al. calculated the maximal information coefficient (MIC), based on the same concept as our calculation of mutual information; MIC was shown to be superior to linear association measures in terms of discovering important relationships [13]. It allows one to capture a wide range of interesting associations, not limited to specific function types, or even to all functional relationships. This generality is very crucial as many important relationships are not well modeled by a function. It was also shown that MIC was equitable in the sense of being able to retain the discovery of various types of associations even with increased noise in the simulation data [13]. These attributes may explain the superior prediction of subject intelligence using network metrics observed in our study. Along similar lines, a study in a cohort of patients with schizophrenia [28] demonstrated that nonlinear functional connectivity provided useful discriminative power toward making the diagnosis in each patient.

This study has several limitations. First, our cohort was a selected population of pediatric patients with focal epilepsy. The results may not be generalizable to other patient populations, or to normal subjects. Second, our sample size was small, which did not allow a study stratified by disease severity or a study on characteristics of patients who benefit more from using mutual information. Nevertheless, our goal was to show the general advantages of a nonlinear method used to quantify functional connections. Finally, the extensive computation time required to generate whole-brain networks under a range of nonlinear methods precludes comparison of an exhaustive list of available methods. Nevertheless, mutual information has been proven to be an effective measure in various disciplines for its generality and equitability.

## 5. Conclusion

Brain networks constructed using edges defined by mutual information significantly outperformed the use of the Pearson correlation for predicting global intelligence in a pediatric cohort with focal epilepsy. Network methodologies specifically optimized to make predictions about individuals will be critical to the development and implementation of clinical tools based on resting-state constructs.

## Figures and Tables

**Table 1:** Metrics of the network architecture.

Metric	Description
Clustering coefficient	The fraction of the nodes of a given neighbor that are also neighbors of each other reflects segregation/subspecialization in the network

Modularity	The degree to which nodes tend to segregate into relatively independent modules reflects segregation/subspecialization within the network

Path length	The minimum number of edges required to traverse the distance between 2 nodes averaged over the network reflects the ease of information transfer across the network

Global efficiency	Inverse of the mean characteristic path length averaged over the network reflects integration in the network

Reproduced from Paldino MJ et al. (2016) [8] (under the Creative Commons Attribution License/public domain).

**Table 2:** Comparison between Pearson and mutual information graph metrics.

	Pearson graph	Mutual information graph	*p* value	*r* (95% CI)
	Mean ± SD	Mean ± SD		
Clustering coefficient	0.26 ± 0.17	0.10 ± 0.08	<0.001	0.88 (0.74, 0.95)
Modularity	0.10 ± 0.04	0.11 ± 0.03	0.246	0.86 (0.68, 0.93)
Path length	3.57 ± 1.14	9.47 ± 3.45	<0.001	0.88 (0.73, 0.94)
Global efficiency	0.35 ± 0.14	0.15 ± 0.08	<0.001	0.84 (0.65, 0.93)

*p* values were adjusted for multiple comparison by the Bonferroni method. SD: standard deviation; *r*: correlation coefficient between Pearson and mutual information graph metrics; CI: confidence interval.

**Table 3:** Association between network metrics and patient IQ.

	Pearson graph		Mutual information graph	
	CC	*p* value	CC	*p* value
Clustering coefficient	−0.56	0.0320	−0.69	0.0016
Modularity	0.53	0.0656	0.52	0.0776
Path length	0.58	0.0232	0.64	0.0056
Global efficiency	−0.57	0.0272	−0.64	0.0064

*p* values were adjusted for multiple comparison by the Bonferroni method. CC: Spearman correlation coefficient between network metric and full-scale intelligence quotient.

**Table 4:** Prediction accuracy for mutual information and Pearson graph metrics.

	Fractional variation explained (95% CI)	Absolute error (mean ± SD)	*p* value of absolute error comparison
Mutual information	49% (46%–51%)	9.1 ± 7.7	0.04
Pearson	17% (13%–19%)	13.0 ± 10.0	

CI: confidence interval; SD: standard deviation.

## Data Availability

The patient characteristics and image data used to support the findings of this study are restricted by the HIPAA in order to protect patient privacy. Data are available from Wei Zhang (wxzhang1@texaschildrens.org) for researchers who meet the criteria for access to confidential data.

## References

[1] Hagmann P., Cammoun L., Gigandet X. (2008). Mapping the structural core of human cerebral cortex. *PLoS Biology*.

[2] Rubinov M., Sporns O. (2010). Complex network measures of brain connectivity: uses and interpretations. *NeuroImage*.

[3] Paldino M. J., Zhang W., Chu Z. D., Golriz F. (2017). Metrics of brain network architecture capture the impact of disease in children with epilepsy. *NeuroImage: Clinical*.

[4] Biswal B. B., Van Kylen J., Hyde J. S. (1997). Simultaneous assessment of flow and BOLD signals in resting-state functional connectivity maps. *NMR in Biomedicine*.

[5] Li Y., Liu Y., Li J. (2009). Brain anatomical network and intelligence. *PLoS Computational Biology*.

[6] van den Heuvel M. P., Stam C. J., Kahn R. S., Hulshoff Pol H. E. (2009). Efficiency of functional brain networks and intellectual performance. *Journal of Neuroscience*.

[7] Kim D. J., Davis E. P., Sandman C. A. (2016). Children’s intellectual ability is associated with structural network integrity. *NeuroImage*.

[8] Paldino M. J., Golriz F., Chapieski M. L., Zhang W., Chu Z. D. (2016). Brain network architecture and global intelligence in children with focal epilepsy. *American Journal of Neuroradiology*.

[9] de Zwart J. A., van Gelderen P., Jansma J. M., Fukunaga M., Bianciardi M., Duyn J. H. (2009). Hemodynamic nonlinearities affect BOLD fMRI response timing and amplitude. *NeuroImage*.

[10] Cassidy B., Rae C., Solo V. (2015). Brain activity: connectivity, sparsity, and mutual information. *IEEE Transactions on Medical Imaging*.

[11] Lahaye P. J., Poline J. B., Flandin G., Dodel S., Garnero L. (2003). Functional connectivity: studying nonlinear, delayed interactions between BOLD signals. *NeuroImage*.

[12] Rummel C., Abela E., Muller M. (2011). Uniform approach to linear and nonlinear interrelation patterns in multivariate time series. *Physical Review E*.

[13] Reshef D. N., Reshef Y. A., Finucane H. K. (2011). Detecting novel associations in large data sets. *Science*.

[14] Smith S. M., Jenkinson M., Woolrich M. W. (2004). Advances in functional and structural MR image analysis and implementation as FSL. *NeuroImage*.

[15] Fonov V., Evans A. C., Botteron K. (2011). Unbiased average age-appropriate atlases for pediatric studies. *NeuroImage*.

[16] Simpson I. J., Cardoso M. J., Modat M. (2015). Probabilistic non-linear registration with spatially adaptive regularisation. *Medical Image Analysis*.

[17] Fischl B., Liu A., Dale A. M. (2001). Automated manifold surgery: constructing geometrically accurate and topologically correct models of the human cerebral cortex. *IEEE Transactions on Medical Imaging*.

[18] Fischl B., Salat D. H., van der Kouwe A. J. (2004). Sequence-independent segmentation of magnetic resonance images. *NeuroImage*.

[19] Thomas C. G., Harshman R. A., Menon R. S. (2002). Noise reduction in BOLD-based fMRI using component analysis. *NeuroImage*.

[20] Bright M. G., Murphy K. (2015). Is fMRI “noise” really noise? Resting state nuisance regressors remove variance with network structure. *NeuroImage*.

[21] Pruim R. H., Mennes M., Buitelaar J. K., Beckmann C. F. (2015). Evaluation of ICA-AROMA and alternative strategies for motion artifact removal in resting state fMRI. *NeuroImage*.

[22] Breiman L. (2001). Random forests. *Machine Learning*.

[23] Pang H., Lin A., Holford M. (2006). Pathway analysis using random forests classification and regression. *Bioinformatics*.

[24] Hlinka J., Palus M., Vejmelka M., Mantini D., Corbetta M. (2011). Functional connectivity in resting-state fMRI: is linear correlation sufficient?. *NeuroImage*.

[25] Gottlich M., Munte T. F., Heldmann M., Kasten M., Hagenah J., Kramer U. M. (2013). Altered resting state brain networks in Parkinson’s disease. *PloS One*.

[26] He H., Sui J., Yu Q. (2012). Altered small-world brain networks in schizophrenia patients during working memory performance. *PloS One*.

[27] Sanz-Arigita E. J., Schoonheim M. M., Damoiseaux J. S. (2010). Loss of ’small-world’ networks in Alzheimer’s disease: graph analysis of FMRI resting-state functional connectivity. *PloS One*.

[28] Su L., Wang L., Shen H., Feng G., Hu D. (2013). Discriminative analysis of non-linear brain connectivity in schizophrenia: an fMRI Study. *Frontiers in Human Neuroscience*.

[29] Widjaja E., Zamyadi M., Raybaud C., Snead O. C., Doesburg S. M., Smith M. L. (2015). Disrupted global and regional structural networks and subnetworks in children with localization-related epilepsy. *American Journal of Neuroradiology*.

[30] Yeo R. A., Ryman S. G., van den Heuvel M. P. (2016). Graph metrics of structural brain networks in individuals with schizophrenia and healthy controls: group differences, relationships with intelligence, and genetics. *Journal of the International Neuropsychological Society*.

[31] DeSalvo M. N., Douw L., Tanaka N., Reinsberger C., Stufflebeam S. M. (2014). Altered structural connectome in temporal lobe epilepsy. *Radiology*.

[32] Liao W., Zhang Z., Pan Z. (2010). Altered functional connectivity and small-world in mesial temporal lobe epilepsy. *PloS One*.

[33] Vlooswijk M. C., Vaessen M. J., Jansen J. F. (2011). Loss of network efficiency associated with cognitive decline in chronic epilepsy. *Neurology*.

[34] Vaessen M. J., Braakman H. M., Heerink J. S. (2013). Abnormal modular organization of functional networks in cognitively impaired children with frontal lobe epilepsy. *Cerebral Cortex*.

[35] Langer N., Pedroni A., Gianotti L. R., Hanggi J., Knoch D., Jancke L. (2012). Functional brain network efficiency predicts intelligence. *Human Brain Mapping*.

[36] Fischer F. U., Wolf D., Scheurich A., Fellgiebel A. (2014). Association of structural global brain network properties with intelligence in normal aging. *PloS One*.

[37] Tijms B. M., Yeung H. M., Sikkes S. A. (2014). Single-subject gray matter graph properties and their relationship with cognitive impairment in early- and late-onset Alzheimer’s disease. *Brain Connectivity*.

[38] Xiang J., Guo H., Cao R., Liang H., Chen J. (2013). An abnormal resting-state functional brain network indicates progression towards Alzheimer’s disease. *Neural Regeneration Research*.

[39] Zhou Y., Yu F., Duong T. (2014). Multiparametric MRI characterization and prediction in autism spectrum disorder using graph theory and machine learning. *PloS One*.

